# Enhanced relativistic-electron beam collimation using two consecutive laser pulses

**DOI:** 10.1038/s41598-019-50401-y

**Published:** 2019-10-01

**Authors:** Sophia Malko, Xavier Vaisseau, Frederic Perez, Dimitri Batani, Alessandro Curcio, Michael Ehret, Javier Honrubia, Katarzyna Jakubowska, Alessio Morace, João Jorge Santos, Luca Volpe

**Affiliations:** 1 Centro de Laseres Pulsados (CLPU), Parque Cientifico, E-37185 Villamayor Salamanca, Spain; 2 University of Salamanca, Salamanca, Spain; 3 Laboratoire pour l’Utilisation des Lasers Intenses, Ecole Polytechnique, CNRS, CEA, UMR 7605, F-91128 Palaiseau, France; 4 Univ. Bordeaux, CNRS, CEA, CELIA (Centre Lasers Intenses et Applications), UMR 5107, F-33405 Talence, France; 5 Laboratori Nazionali di Frascati (INFN), Frascati, 00044 Italy; 6 Institut für Kernphysik, Technische Universität Darmstadt, Schlossgartenstrasse 9, 64289 Darmstadt, Germany; 7 ETSI Aeronáuticos, Universidad Politécnica de Madrid, Madrid, Spain; 8 Institute of Plasma Physics and Laser Microfusion, Hery 23, 01-497 Warsaw, Poland; 9 Institute of Laser Engineering, Osaka University, 2-6 Yamadaoka, Suita, Osaka, 565-0871 Japan; 10 Laser-Plasma Chair at the University of Salamanca, Salamanca, Spain

**Keywords:** Laser-produced plasmas, Imaging techniques

## Abstract

The double laser pulse approach to relativistic electron beam (REB) collimation in solid targets has been investigated at the LULI-ELFIE facility. In this scheme two collinear laser pulses are focused onto a solid target with a given intensity ratio and time delay to generate REBs. The magnetic field generated by the first laser-driven REB is used to guide the REB generated by a second delayed laser pulse. We show how electron beam collimation can be controlled by properly adjusting the ratio of focus size and the delay time between the two pulses. We found that the maximum of electron beam collimation is clearly dependent on the laser focal spot size ratio and related to the magnetic field dynamics. Cu-K_*α*_ and CTR imaging diagnostics were implemented to evaluate the collimation effects on the respectively low energy (≤100 keV) and high energy (≥MeV) components of the REB.

## Introduction

The study of the transport of relativistic laser-driven electrons is a subject of interest for many applications including proton-ion acceleration^1–5^, fast ignition approach to inertial confinement fusion (ICF)^6–9^, astrophysics applications^10^, isohoric heating of matter^11–14^ as well as high brilliance and compact laser-based x-ray sources^15,16^. Reducing electron beam divergence^17^ as well as optimizing electron beam transport in plasmas is crucial for several important application: i) In proton acceleration induced by laser it has been proved that a transversal confinement of the electron beam increases the maximum energy of the proton beam; ii) In the fast ignition approach to ICF electron beam collimation is crucial for the success of the scheme. Previous investigations have shown that the dynamics of electron beams propagation in plasmas is mainly affected by: i) resistivity effects^13,18–25^ on the electron stopping power, which become important at relativistic intensities (${I}_{L}\ge {10}^{18}\,{\rm{W}}.{{\rm{cm}}}^{-2}$) and reduce the final penetration length of the electron beam; ii) collisionless Weibel instabilities which start to grow and become very important for laser intensities ${I}_{L} > {10}^{19}\,{\rm{W}}.{{\rm{cm}}}^{-2}$, at the level of the plasma skin depth, generating micro magnetic fields that strongly contribute to increase the initial electron divergence^26^. Different strategies to control REB propagation in solid matter have been proposed. They rely on the use of ~kT magnetic fields, which can be externally generated by coils^27,28^ or self generated^29^, either by artificial resistivity gradients^30–34^ or by exploiting the intrinsically high resistivity of a material^35^. One of the schemes by using self-generated magnetic fields was proposed by A. Robinson *et al*.^36,37^. In this scheme two collinear laser pulses (1 and 2) with a given intensity ratio ${I}_{2}/{I}_{1}\sim 10$ separated by a delay ($\Delta t={t}_{2}-{t}_{1}$) are used to generate fast electron beams. The electron beam produced by the first, less intense, laser pulse generates a resistive azimuthal magnetic field (seed magnetic field) which is used to guide the main electron population generated by the second beam. This scheme was experimentally investigated by Scott *et al*.^38^ who have shown the existence of an optimum delay between the laser pulses of the order of the laser pulse duration ($\Delta t\sim \tau$), at which a maximum electron beam collimation is reached. The existence of this optimum can be explained by considering the growth rate and then the dynamics of the spatial diffusion of the seed magnetic field, in connection with the arrival time of the main electron beam. However the reported study^38^ was focused only on the influence of the delay time between the laser pulses while other relevant parameters, namely laser intensity and laser focal spot size ratio, were kept constant. The condition for collimation suggested by Robinson *et al*.^36^ (by assuming the Larmor radius of the second beam smaller than the radial extension of the seed magnetic field) written in terms of the laser focal spots ratio ${\phi }_{1} > {\phi }_{2}$ was satisfied.

Numerical investigations have been performed to better understand the mechanism^39^. From this study a clearer picture of the physical mechanism is obtained suggesting the relevant role of the ratio between the REB sizes. Following such interpretation we have performed an experimental campaign in which we used two independent focusing parabolic mirrors, allowing to vary the ratio ${\phi }_{1}/{\phi }_{2}$ between the two laser focal spots, therefore controlling the ratio between the radius of the azimuthal magnetic field created by the first beam *R*_1_ and the radius of the second electron beam *R*_2_. In addition to the Cu-K_*α*_ emission diagnostic used in^38^ and mainly sensitive to the more numerous electrons in the 10–100 keV range, we implemented measurement of coherent transition radiation (CTR) to evaluate the collimation effect on higher energy electrons ($\gtrsim$1 MeV)^40^. The performed experimental study with various laser parameters allowed us to make a detailed characterization of the collimation efficiency.

## Results

### Experimental results

**Figure 1:**
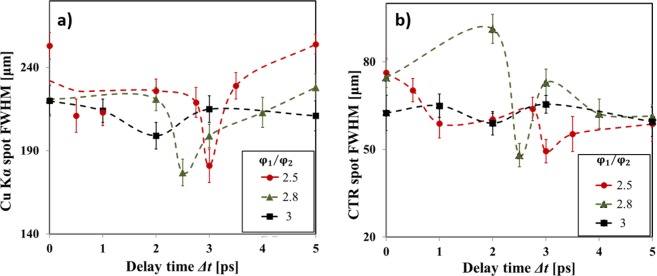
Evolution of the diameter of the emission area on target rear side of (**a**) Cu-K_*α*_ fluorescence and (**b**) CTR, as a function of the delay between the two laser pulses for different focal spot ratios: ${\phi }_{1}/{\phi }_{2}=2.5$ (red circles), ${\phi }_{1}/{\phi }_{2}=2.8$ (green triangles), ${\phi }_{1}/{\phi }_{2}=3$ (black squares). The dashed curves are guides for the eyes. Different vertical scales are used in (**a)** and **(b**).

**Figure 2:**
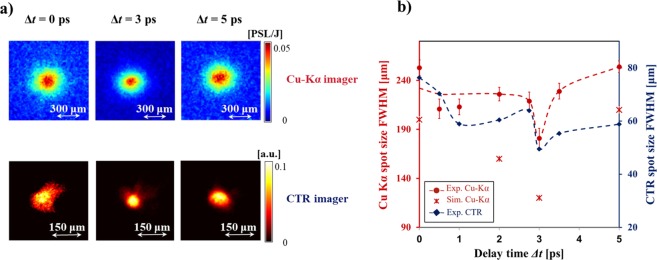
Data obtained for a focal spot ratio ${\phi }_{1}/{\phi }_{2}=2.5$. (**a**) Set of typical Cu-K_*α*_ (top) and CTR (bottom) images obtained at different delays Δ*t* = 0 ps (left), 3 ps (middle) and 5 ps (right). (**b**) Comparison of Cu-K_*α*_ (red circles) and of CTR (blue circles) emission spot sizes. The red crosses show the results of the simulated Cu-K_*α*_ emission, reproducing the delay at which optimal collimation occurs.

Figure 1(a) presents the evolution of the Cu-K_*α*_ spot size as a function of delay time between the two laser pulses for different ratios ${\phi }_{1}/{\phi }_{2}$. An optimum delay, corresponding to a maximum collimation of the fast electron beams, was measured for each focal spot ratio. Both Cu-K_*α*_ and CTR diagnostics confirm that the collimation of the main electron beam occurs at delays $\Delta t=3,2.5,2\,{\rm{ps}}$ respectively for the run with focal spot ratios ${\phi }_{1}/{\phi }_{2}=2.5,2.8,3$ [Fig. 1(b)]. The higher the ratio ${\phi }_{1}/{\phi }_{2}$, the shorter is the delay at which an optimum collimation occurred. Examples of experimental images are shown in Fig. 2(a) for ${\phi }_{1}/{\phi }_{2}=2.5$, while Fig. 2(b) reports the size as measured by the two diagnostics on the same graph [same data as Fig. 2(a)]. Compared to the K_*α*_ signals the absolute smaller size of CTR signal confirms that this emission is due to the high energy component of electron beam, which has a smaller angular spread. Efficient electron beam collimation can be represented by introducing the compression parameter C defined as the ratio between the Cu-K_*α*_ peak intensity and the Cu-K_*α*_ spot FWHM^39^. A compression of the beam is indeed achieved when a reduction of the electron beam size is accompanied by an increase of the peak intensity of the signal: a larger value of C corresponds to a more collimated electron beam. The maximum compression corresponds to the maximum value of Cu-K_*α*_ emission at the delay time of 3 ps where the electron beam area is decreased by a factor of 0.5 and the Cu-K_*α*_ intensity is increased by a factor of 1.37 [Fig. 4]. This suggests that more than 70% of hot electrons are collimated in the process. The CTR signal shows also a reduction of the beam size by a fairly comparable factor ~0.6 although there is not a clear increase of the detected signal yield. This seems to suggest a lower effect of the magnetic field on the high energy electron beam component which results both because of the larger difference between the radial extent of the magnetic field and the spatial size of the high energy component in the beam, and the smaller deviation of higher energy electrons.

**Figure 3:**
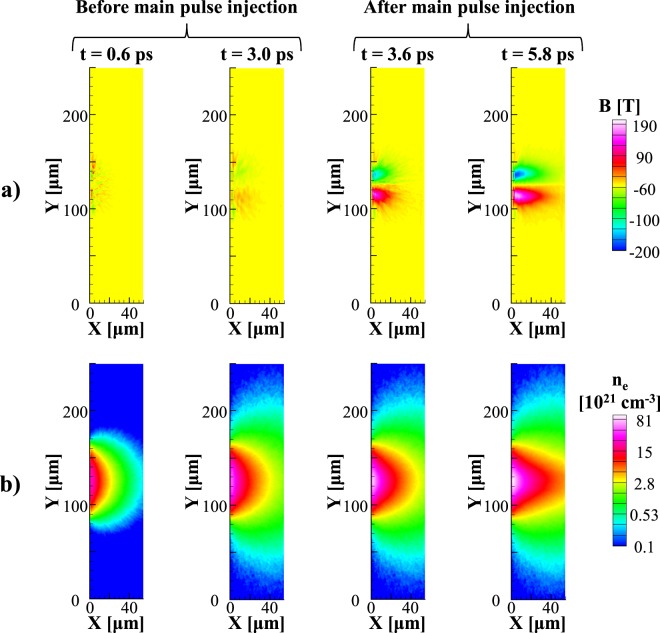
Results of fast electron transport simulations for the focal spot ratio ${\phi }_{1}/{\phi }_{2}=2.5$ and and for a delay time between the two laser pulses Δ*t* = 3 ps. (**a**) Evolution of the azimuthal magnetic field Y component and (**b**) of the fast electron density extracted at t = 0.6 ps, 3 ps (before main pulse injection) and at t = 3.6 ps, 5.8 ps (after main pulse injection).

**Figure 4:**
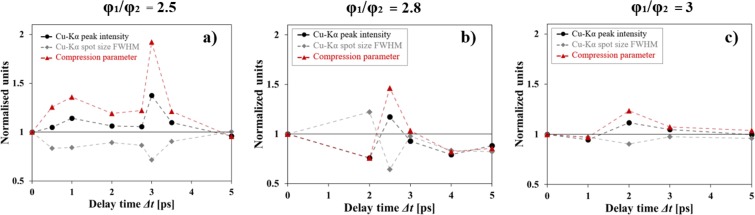
Evolution of the Cu-K_*α*_ peak intensity (black circles), Cu-K_*α*_ emission spot size (grey circles) and compression factor C (red triangles), normalized to the values at Δ*t* = 0 ps for the run with the focal spot ratio ${\phi }_{1}/{\phi }_{2}=2.5$ (**a**), ${\phi }_{1}/{\phi }_{2}=2.8$ (**b**), ${\phi }_{1}/{\phi }_{2}=3$ (**c**).

### Simulations

In order to support our physical interpretation of the experimental results of fast electron collimation, we performed a set of numerical simulations covering all the involved time scales. The plasma expansion at the target front surface was estimated using the 1D hydro code MULTI^41^ while the laser-driven electron source was characterized using the 2D Particle-in-Cell (PIC) code SMILEI^42^. The transport of fast electrons through the target was simulated over the time $t=3\,{\rm{ps}}+\Delta t$ using a 3D hybrid-PIC code^43,44^, reproducing the experimental configuration for the run with a laser focal spot ratio ${\phi }_{1}/{\phi }_{2}=2.5$.

Results of hybrid simulations are compared with the experiment in terms of Cu-K_*α*_ emission spot sizes. As shown in Fig. 2(b) both the experimental and the synthetic Cu-K_*α*_ spot size exhibit a two times decrease at optimum delay when compared to a simultaneous shot of the two laser beams ($\Delta t=0$). The minimum spot size is also reached with a delay time ~3 ps, when the amplitude of magnetic field reached its maximum *B*_*max*_~200 T [Fig. 3(a)]. It is worth to note that the discrepancy in terms of size between the simulated and the experimental spots might be related to an underestimation of the fast electron beam divergence and the fast electron beam diameter.

## Discussion

Two main effects were observed when varying the laser focal spot ratio of the laser pulses: a variation of the maximum compression coefficient and a shift of the optimum delay time. Such tendencies are explained by the dynamics of the self-generated magnetic fields governed by the diffusion equation which combines the generalized Ohm’s law^45,46^ with Maxwell-Faraday’s law:

1 $$\frac{\partial \overrightarrow{B}}{\partial t}=\eta \overrightarrow{\nabla }\times {\overrightarrow{j}}_{b}+\overrightarrow{\nabla }(\eta )\times {\overrightarrow{j}}_{b}+\frac{\eta }{{\mu }_{0}}{\overrightarrow{\nabla }}^{2}\overrightarrow{B}-\frac{1}{{\mu }_{0}}\overrightarrow{\nabla }(\eta )\times \overrightarrow{B}$$

with the plasma resistivity $\eta$, the magnetic field *B* and the fast electron current density *j*_*b*_. The terms in the right-hand-side of Eq. 1 are responsible for the magnetic field generation and evolution. The maximum amplitude *B*_*max*_, the rise and diffusion times are mainly dependent on the laser pulse duration, intensity and focal spot size via the target resistivity evolution and the fast electron beam current density. Applying this equation to our case, we can explain the magnetic field dynamics and its influence on the observed electron beam collimation. An increase of the focal spot size of the first laser pulse causes (see Fig. 4):

1. A reduction of the optimum delay time
2. An increase of the time window for second electron beam injection
3. A mitigation of the REB collimation.

The later effect (3), estimated by the compression ratio, is caused by the natural reduction of the maximum amplitude of the magnetic field *B*_*max*_ because a larger spot implies a reduced laser intensity on target: the Δ*t* scan with ${\phi }_{1}/{\phi }_{2}=3$ is the less efficient. The reduction of the optimum delay time between the two laser pulses when the focal spot ratio increased (1) is due to the change in target resistivity following the evolution of the target temperature. With the increase of laser focal spot ${\phi }_{1}$, the injected energy density reduces, therefore the target electron temperature *T*_*e*_ decreases and the resistivity $\eta$ gets larger implying a decrease of the B-field rise time $\xi \sim \frac{1}{\eta }$. As a consequence the magnetic field reaches *B*_*max*_ faster, when the REB collimation is observed. As for the optimum time window (2) for the injection of the second electron beam, this appears because the collimation of the REB is caused by a resistive magnetic field presenting a sufficiently long lifetime, the later being directly related to the magnetic field diffusion time, scaling as ${\tau }_{diff}\propto \frac{{R}^{2}}{\eta }$ [Eq. 1]. As a consequence, the bigger the radial size of the first electron beam, the longer the seed magnetic field lasts, extending the optimum time window for the injection of the main electron population. The existence of the optimum focal spot ratio, when the compression reaches its maximum, is a trade-off between the maximum amplitude of the magnetic field *B*_*max*_ and its diffusion time. The laser focal spot ratio should lay between 2–2.8, the most evident collimation effect having been observed for a focal spot ratio of 2.5. This is based on the experimental measurements for three focal spot ratio cases and the limitation of the scheme working condition caused by laser pointing issues. It is worth to note that one of the crucial requirements for this experimental campaign is the necessary overlapping of the laser focal spots. Increasing the focal spot of the seed laser pulse guarantees a better spatial overlapping of the two laser focal spots. A focal spot ratio smaller than 2 would have decreased the probability of overlapping the focal spots from one shot to another.

In summary, in the present experiment, we extensively studied the double pulse approach to the collimating of relativistic electron beams produced in high-intensity laser-plasma interactions. By changing the ratio between the focal spots of the two lasers ${\phi }_{1}/{\phi }_{2}$ and the injection time Δ*t*, we observed a clear signature of collimation, validating the theory presented in^36,39^. Two complementary diagnostic techniques have been implemented, which mainly show the respective behaviour of very fast vs. less fast hot electrons. The results are essentially in agreement. In particular, both from experimental results and from simulations, we have shown that for each value of ${\phi }_{1}/{\phi }_{2}$ there is an optimal injection time Δ*t*, which, in agreement with expectations, increases when ${\phi }_{1}/{\phi }_{2}$ is decreased. We also found that about 70% of hot electrons can be collimated by this mechanism. In conclusion, the double pulse technique appears to be an easy and controllable way to limit the divergence of fast electrons and improve energy transport deep into the target. This result opens interesting perspectives for a large variety of applications including the fast ignition approach to inertial confinement fusion and the optimisation of laser-driven particle sources.

## Methods

### Experimental set-up and diagnostics

**Figure 5:**
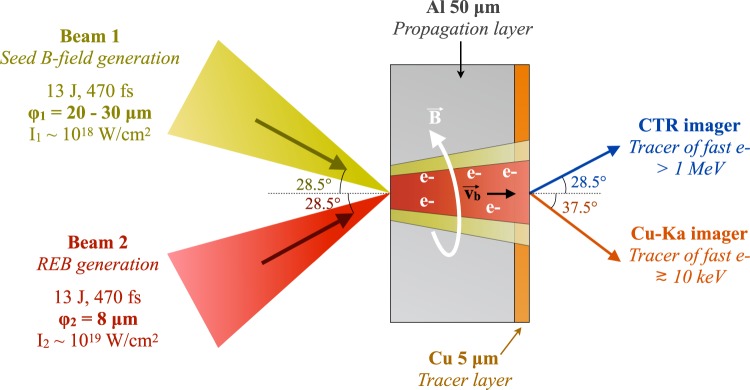
Experimental setup.

The experiment was performed at the LULI-ELFIE facility (Ecole Polytechnique, France). We used a dual beam configuration, with two *λ* = 1.06 *μ*m, *τ* = 470 fs full-width-at-half-maximum (FWHM) pulses, containing 13 ± 2 J of energy each and focused symmetrically at ± 28.5° incidences with respect to the target normal [Fig. 5]. The use of two different off-axis parabolic mirrors, one for each beam, allowed to vary the focal spot size of the first pulse, generating the seed magnetic field, from ${\phi }_{1}=20$ *μ*m to 30 *μ*m FWHM while keeping constant the focal spot of the second pulse (${\phi }_{2}=8$ *μ*m FWHM). These yielded intensities of ~10^18^ W.cm^−2^ and 1 × 10^19^ W.cm^−2^, respectively. The 3 × 3 mm^2^ planar double-layer targets were composed of Al[50 *μ*m] − Cu[5 *μ*m], with the Al layer facing the two laser pulses. The two pulses, originating from the same oscillator, were temporally synchronized using interferometry techniques. The delay Δ*t* between the laser pulses was varied between 0 ps and 5 ps, with a precision of 100 fs. Cu-K_*α*_ x-ray emission ($\hslash \omega \approx 8\,{\rm{keV}}$) produced by electrons passing through the copper tracer was imaged onto a FUJI image plate using a spherically bent quartz 22–43 crystal^16^, with a radius of curvature of 25 mm, looking at 37.5° with respect to the target normal. Coherent transition radiation at twice the laser frequency^47^ produced by relativistic electrons of energies $\gtrsim$1 MeV was recorded using a Gated Optical Imager (GOI) looking at the target rear surface at 28.5° with respect to the target normal and with an acquisition time of 200 ps, limiting the contribution to the signal of delayed Planckian thermal radiation.

### Numerical simulations

As stated above in order to support our physical interpretation of the experimental results of fast electron collimation, we performed a set of numerical simulations. The pre-plasma formation by the interaction of the seed laser pulse with the front side aluminium layer was evaluated using the hydrodynamic code MULTI^41^ in 1D. The plasma electron density profile showed an approximately exponential profile which could be fitted as ${n}_{e}(x)\propto {e}^{\frac{3}{2}x}$, where *x*[*μm*] is the longitudinal coordinate. The parameters of the electron source produced by the interaction of the main laser pulse with a 50 *μ*m thick aluminium layer were evaluated via Particle-in-Cell (PIC) simulations in 2D using the SMILEI code^42^. We considered a 470 fs FWHM Gaussian pulse with 10^19^ W.cm^−2^ peak intensity. The extracted REB energy distribution was averaged over the 1.5 ps duration of the simulation and is well described in the 10 keV ≤ *E* ≤ 200 MeV energy range by the following analytical expression: $f(E)=\exp (\,-\,\frac{E}{{T}_{b}})+(\frac{{T}_{c}}{E})\times {[\frac{({\gamma }_{0}-1){m}_{e}{c}^{2}}{E}]}^{a}\,\exp \,(\,-\,\frac{E}{{T}_{sh}})$. The fitting parameters are: ${T}_{b}=30.3\,\,{\rm{keV}}$, ${T}_{sh}=10\,\,{\rm{MeV}}$, ${T}_{c}=1\,{\rm{MeV}}$, ${\gamma }_{0}=1.0075$, $a=1.6$. The transport of fast electrons into the target was simulated in 3D with a hybrid-PIC code^43,44^ using the aforementioned electron distribution as input to reproduces the experimental configuration for the run with a laser focal spot ratio ${\phi }_{1}/{\phi }_{2}=2.5$ over the time $t=3\,{\rm{ps}}+\Delta t$. The laser-to-fast-electrons conversion efficiency was set to 25% according to^48^. The electric resistivity is calculated using the Eidmann-Chimier model^49,50^. The fast electron angular distribution is fitted by the function $f(\theta ,r,E)\propto \exp [{(-\frac{\theta -{\theta }_{r}}{\Delta \theta })}^{2}]$, where Δ*θ* is the dispersion angle at the source and *θ*_*r*_ = arctan[tan(*γ*)*r*/*r*_0_] is the mean radial angle with respect to the laser propagation axis with *r*_0_ = 13.5 *μ*m. The angles were estimated according to^17^ as Δ*θ* = 45° and *γ* = 35° for the first electron beam, and 55° and 45° for the second electron source respectively.
